# Headache Secondary to Sphenoid Sinus Fungal Mycetoma

**DOI:** 10.7759/cureus.79561

**Published:** 2025-02-24

**Authors:** Mohd Iqbal Mohd Ayob, Farah Dayana Zahedi, Mohd Zambri Ibrahim

**Affiliations:** 1 Department of Otorhinolaryngology - Head and Neck Surgery, Universiti Kebangsaan Malaysia Medical Centre, Kuala Lumpur, MYS; 2 Department of Otorhinolaryngology, Hospital Tuanku Fauziah, Kangar, MYS

**Keywords:** fungal, fungal ball, headache, mycetoma, sphenoid sinus

## Abstract

Sphenoid sinus fungal ball is a rare occurrence. To diagnose isolated sphenoid sinus fungal ball pathology accurately and promptly, a high index of clinical suspicion, routine office nasal endoscopy, and radiological imaging are essential. In this case report, we discuss a woman in her 60s who presented with chronic generalized headache. Incidental findings of computed tomography of the brain revealed a lesion in the sphenoid sinus, which was later diagnosed and treated as a fungal mycetoma.

## Introduction

Humans are consistently exposed to fungal spores in both indoor and outdoor environments. We inhale these spores regularly, and they settle along the mucosa of the respiratory airways. While certain fungal infections are opportunistic, others function as true pathogens. Various factors, including the body's temperature of 37°C, the low redox potential of tissues, and the effectiveness of our immune system, act as barriers preventing the majority of fungi from invading the human host [1].

Due to the anatomical features of the sphenoid sinus, individuals may manifest with diverse nonspecific symptoms and complications. The predominant initial symptom is typically a unilateral headache corresponding to the side of the lesions. While pain relievers initially prove effective in managing discomfort during the early stages of the condition, their efficacy diminishes as the disease progresses [2].

Generally, there are two types of fungal sinusitis: invasive and noninvasive. It is based on whether microscopic evidence of fungal hyphae is present in the tissues, such as the blood vessels, bone, or mucosa. In noninvasive fungal sinus diseases, the prevalence of fungal ball cases has been increasing, with extensive published studies enhancing our understanding of the condition and guiding the development of effective treatment strategies [3].

## Case presentation

An elderly woman with underlying hypertension and hepatitis C presented with a generalized throbbing headache lasting one month, which worsened over the last three weeks. It was aggravated by coughing and sneezing. The pain score was 6/10 on the numeric pain rating scale and was only partially alleviated with paracetamol. Otherwise, there were no symptoms suggestive of increased intracranial pressure, such as vomiting or blurred vision. There was no nasal discharge, congestion, or hyposmia. She also had no systemic symptoms such as fever, lethargy, or malaise.

Upon general examination, the patient appeared well and had stable vital signs. Nasoendoscopic examination revealed a left septal spur touching the inferior turbinate. The turbinates, osteomeatal complexes, fossa of Rosenmüller, and nasopharynx were normal.

**Figure 1:**
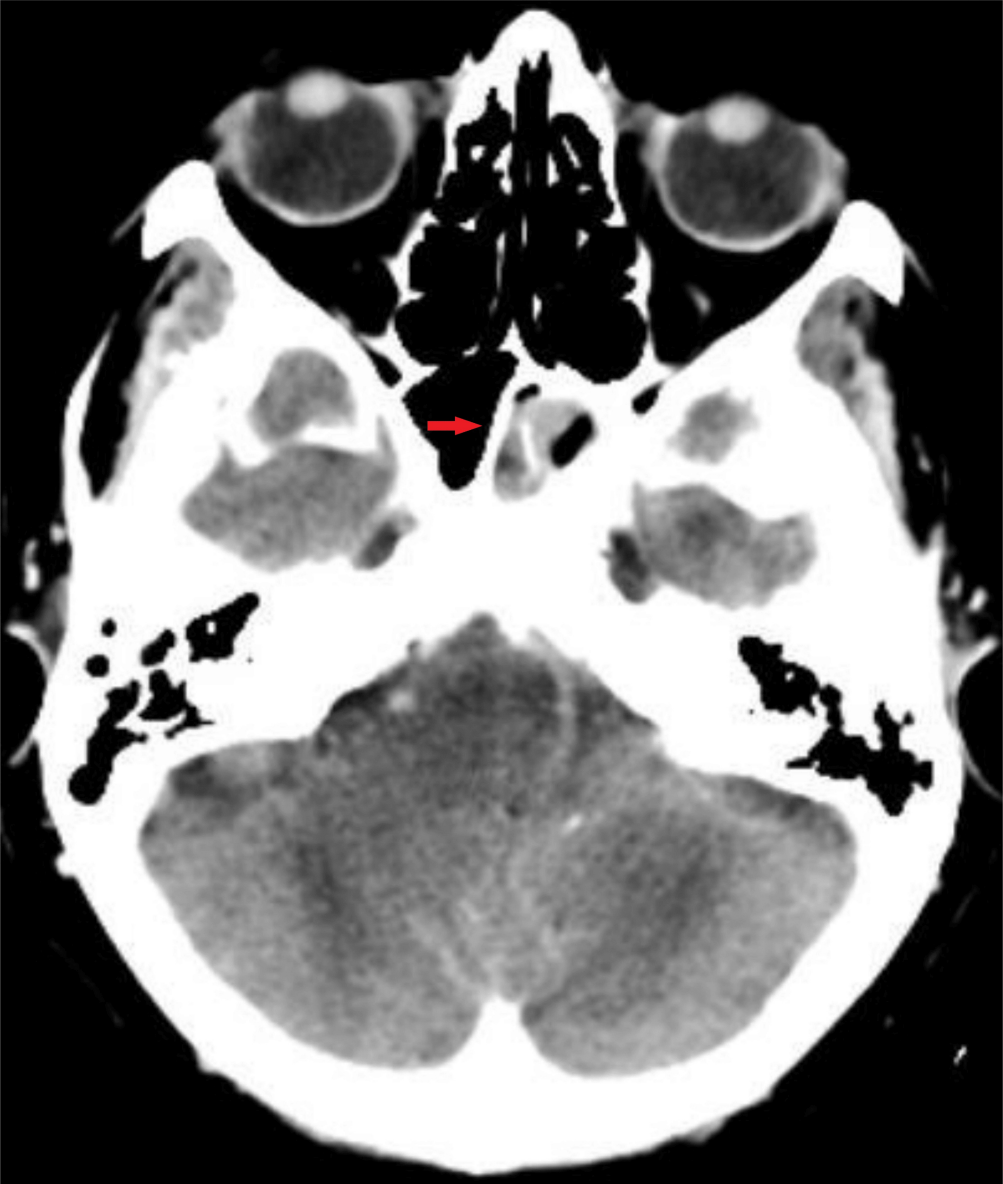
Axial view of the contrast-enhanced CT scan of paranasal sinus. The red arrow shows mucosal thickening of the left sphenoid sinus. The lesion shows enhancement from the rest of the mucosa with a calcified deposit within it. There is abnormal erosion on the floor of sella turcica CT: computed tomography

**Figure 2:**
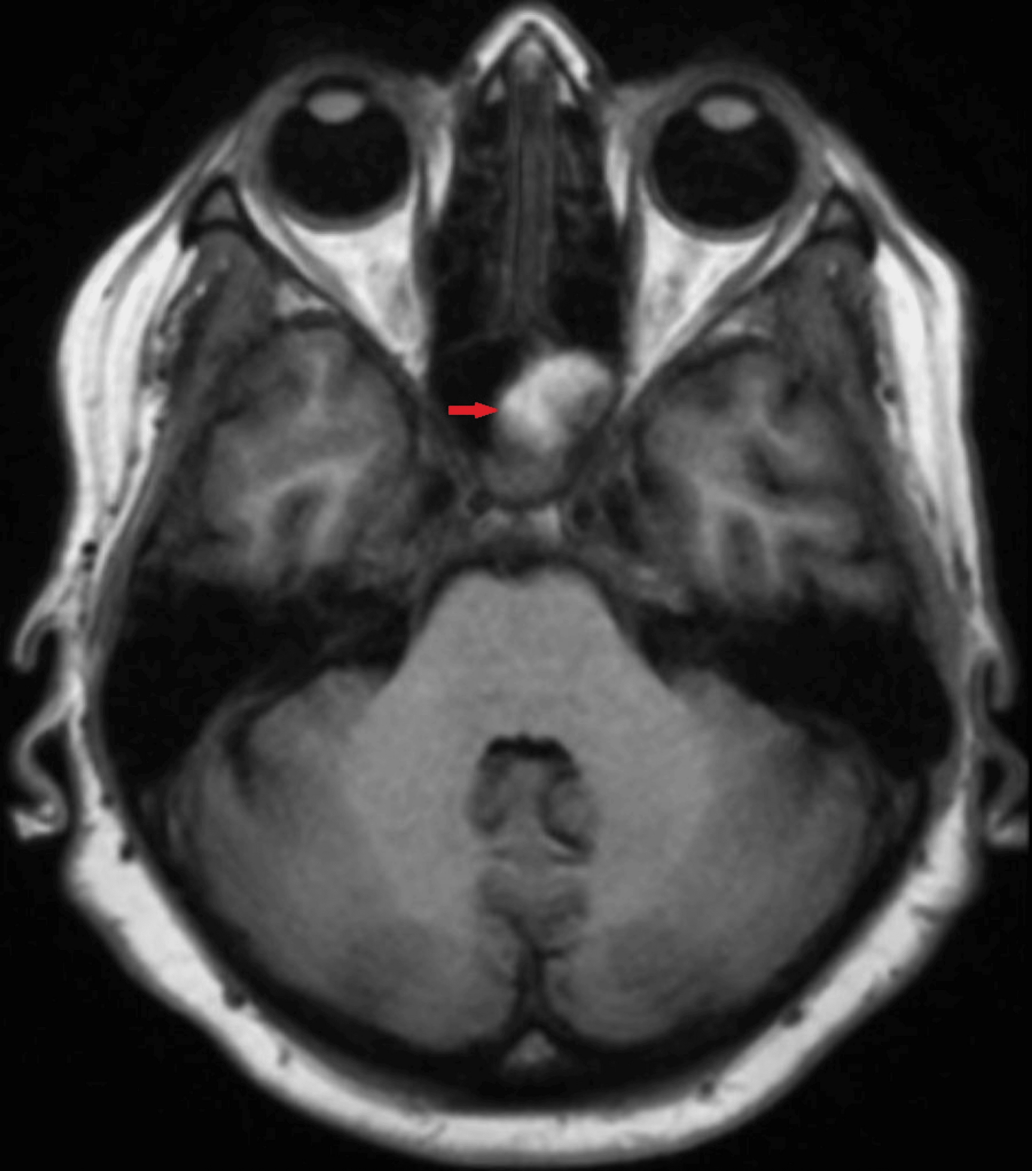
Magnetic resonance imaging, T2-weighted axial view. The red arrow indicates the sphenoid sinus, which demonstrates T1 hyperintensity along with a blooming artifact, without any expansion of the sinus

A computed tomography (CT) scan of the paranasal sinus and magnetic resonance imaging (MRI) of the head and neck were performed. Notable findings from the CT scan of the paranasal sinus included total opacification of the left sphenoid sinus, with a rim of calcification and a few calcified deposits within it suggestive of a fungal ball. There was also erosion of the floor of the sella turcica, as seen in Figure 1. Other bony areas were intact, and the brain parenchyma appeared normal. The MRI of the head and neck showed a T1 hyperintense and T2 hypointense soft tissue lesion occupying the left sphenoid sinus, with a blooming artifact, as seen in Figure 2.

**Figure 3:**
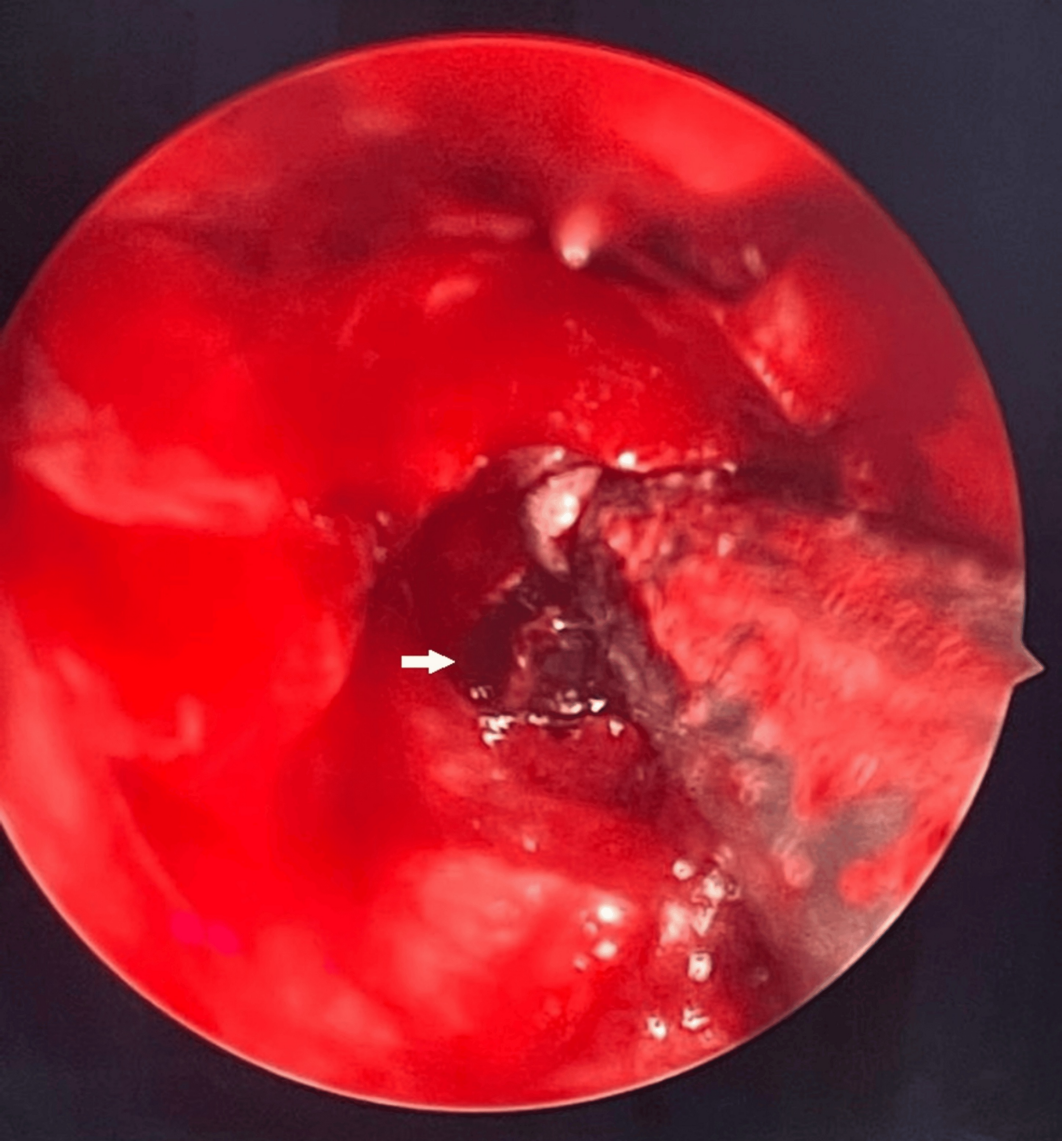
Intraoperative view of left sphenoid sinus. White arrow shows blackish material within the sphenoid sinus

She underwent endoscopic ethmoidectomy, sphenoidotomy, and septoplasty. Intraoperative findings included blackish material within the left sphenoid sinus, which was completely removed and sent for histopathological examination, as seen in Figure 3. The wall of the left sphenoid sinus was thickened anteriorly, and no erosion was seen. The histopathological examination result was a fungal mycetoma. She was discharged well after the operation.

## Discussion

A fungal mycetoma refers to a noninvasive, concentrated gathering of fungal hyphae within a single sinus cavity. Histologically, fungal balls exhibit entangled masses of fungal organisms or fungi embedded in fibrinous, necrotic exudate with minimal mucosal inflammatory response. Typically, this condition is unilateral, affecting only one sinus in most cases (up to 90%-99%), with the maxillary sinus being the most commonly affected [4,5]. The second most common location for this disorder is the sphenoid sinus. Sphenoid fungal mycetoma is rare in clinical practice in comparison to other sites of the disease (56/226; 24.78%) [6]. In 2009, Leroux et al. reported 24 cases in 10 years, whereas Nicolai et al. documented 23 cases in 16 years [7,8]. Interestingly, it is more common in older women, with a male-to-female ratio of 1:2.3 (73.2%) with a mean age of 62 years. The underlying mechanism for its higher occurrence in elderly women has not yet been identified. The pathophysiology of fungus balls has not been clearly understood, but one of the most likely causes is impairment of sinus ventilation.

Depending on the affected sinus, a mycetoma will present differently clinically. The symptomatology can be acute or chronic [9]. Nasal symptoms such as cacosmia, mucopurulent discharge, postnasal drip, and congestion are commonly present in maxillary and ethmoid mycetoma. On the affected side, facial tenderness is additional supportive evidence [10].

Headache is the most common symptom of sphenoid sinus mycetoma [6,7,11-13]. Sphenoid sinus disease should be considered a potential diagnosis in patients experiencing acute or subacute headaches. Numerous descriptions of headache localization can be found in the literature, including periorbital, retro-orbital, occipital, frontal, and diffuse headaches [7,13-15]. Other literature has reported headaches, with the most prevalent locations being the retro-orbital and occipital regions [12,16]. Headache in sphenoid sinus fungal mycetoma does not have a specific pathognomonic area. In other words, the location of the headache may not directly correspond to the affected sinus. In this case, our patient had been having headaches on and off for the past one month and worsening for the last three weeks, which was aggravated by coughing and sneezing. The headache was generalized.

Headaches may arise from pressure on the sphenoid cavity when the sinus is fully filled, dural inflammation caused by sphenoid wall erosion, or irritation of the ophthalmic (V1) and maxillary (V2) branches of the trigeminal nerve that supply the sphenoid sinus [17].

CT and MRI are crucial imaging tools for identifying sinus inflammation and ruling out other brain abnormalities. It is essential to consider various differential diagnoses, including bacterial sinusitis, mucoceles, malignant tumors, and metastases [18]. CT imaging is frequently used to detect sphenoid disease, including fungal balls that present as opacities with central microcalcifications (Figure 1). Metal-dense hyperintensities, attributed to the presence of iron or manganese within a fungus ball, are highly predictive of fungal infection [19]. MRI may also be useful in identifying a fungal ball from a mucocele, benign tumors, encephaloceles, and internal carotid artery aneurysm. It usually appears as a nonenhancing hypointense mass on a T1-weighted image, but the presence of tiny calcifications within the lesion may confer a hyperintense signal with a blooming effect (Figure 2) [20].

## Conclusions

Sphenoid fungal ball is a rare disease that sometimes has vague symptoms. It should be suspected in patients experiencing unexplained headaches. CT scan is the most common imaging investigation for diagnosis, and MRI may provide further information to evaluate the extracompartmental invasion. Furthermore, given the sphenoid sinus's crucial anatomical location, early treatment of sphenoid sinus mycetoma is essential to prevent further damage or spread to adjacent structures.
